# EEVD motif of heat shock cognate protein 70 contributes to bacterial uptake by trophoblast giant cells

**DOI:** 10.1186/1423-0127-16-113

**Published:** 2009-12-15

**Authors:** Kenta Watanabe, Masato Tachibana, Suk Kim, Masahisa Watarai

**Affiliations:** 1 Department of Veterinary Public Health, Faculty of Agriculture, Yamaguchi University, Yamaguchi 753-8515, Japan; 2 Department of Veterinary Public Health, Gyeongsang National University, Gyeongnam 660-701, Korea

## Abstract

**Background:**

The uptake of abortion-inducing pathogens by trophoblast giant (TG) cells is a key event in infectious abortion. However, little is known about phagocytic functions of TG cells against the pathogens. Here we show that heat shock cognate protein 70 (Hsc70) contributes to bacterial uptake by TG cells and the EEVD motif of Hsc70 plays an important role in this.

**Methods:**

*Brucella abortus *and *Listeria monocytogenes *were used as the bacterial antigen in this study. Recombinant proteins containing tetratricopeptide repeat (TPR) domains were constructed and confirmation of the binding capacity to Hsc70 was assessed by ELISA. The recombinant TPR proteins were used for investigation of the effect of TPR proteins on bacterial uptake by TG cells and on pregnancy in mice.

**Results:**

The monoclonal antibody that inhibits bacterial uptake by TG cells reacted with the EEVD motif of Hsc70. Bacterial TPR proteins bound to the C-terminal of Hsc70 through its EEVD motif and this binding inhibited bacterial uptake by TG cells. Infectious abortion was also prevented by blocking the EEVD motif of Hsc70.

**Conclusions:**

Our results demonstrate that surface located Hsc70 on TG cells mediates the uptake of pathogenic bacteria and proteins containing the TPR domain inhibit the function of Hsc70 by binding to its EEVD motif. These molecules may be useful in the development of methods for preventing infectious abortion.

## Background

The placenta is a dynamic organ consisting of maternal and fetal tissues, forming an impermeable physical and biological barrier that protects the fetus against pathogens [1,2]. Several intracellular pathogens can cross this barrier and succeed the vertical transmission. These include some bacteria such as *Brucella abortus*, *Chlamydophila psittaci*, *Coxiella burnetii*, and *Listeria monocytogenes *[1], viruses such as cytomegalovirus and parvovirus B19 [3], and parasites such as *Toxoplasma gondii *[2]. However, the precise molecular mechanisms of the vertical transmission of these pathogens are still unclear.

Brucellosis is a widespread and economically important infectious disease of humans and animals caused by members of the genus *Brucella*. *Brucella *spp. are small, gram-negative, facultative intracellular pathogens that cause abortion, and are retained in the placenta and causing infertility in numerous domestic and wild mammals. In humans they cause undulant fever [4]. Infection in humans is almost exclusively due to zoonosis, either through direct contact with infected animals or from contaminated dairy products [4]. *L. monocytogenes *is a gram-positive bacterium found widely in nature. As a facultative intracellular food-borne pathogen, it is responsible for both severe central nervous system and fetal infections in humans and in a large variety of animals [5]. Although human listeriosis occurs anytime during pregnancy, it is frequently detected during the third trimester, resulting in intrauterine fetal death, abortion, preterm birth, or neonatal infection with a severe septic syndrome known as granulomatosis infantiseptica.

The infectious abortion model using pregnant mice is a powerful tool for investigating the mechanisms of pathogen infection. In our previous study, we demonstrated that *B. abortus *causes abortion in pregnant mice by inoculating bacteria on day 4.5 of gestation [6,7]. We found that there was a higher degree of bacterial colonization in the placenta than in other organs, that there were many bacteria in trophoblast giant (TG) cells in the placenta and that an intracellular replication-defective mutant did not induce abortion. These findings suggest that bacterial infection of TG cells plays a key role in abortion induced by *B. abortus *infection. Also, several studies have reported on an experimental model of listeriosis using pregnant mice [8-10]. Trophoblastic cells are the early targets of *L. monocytogenes *and bacteria then disseminate rapidly to the other trophoblastic structures, such as the syncytiotrophoblast cells lining the villous core in the labyrinthine zone of the placenta [10]. Despite some aspects unique to rodents, notably blood circulation [11], the mouse placenta is comparable to that of humans in that both are hemochorial placentas [12]. It is known that fetal-embryonic trophoblast cells play a central role in the development and physiology of the placenta, including the establishment of local immunotolerance [9]. This structure also has an area of high phagocytic activity [13].

In mice and other rodents, TG cells are the placental cells in direct contact with endometrial tissues throughout gestation [14]. After the onset of implantation, the phagocytosis of maternal components develops in TG cells. It has been reported that this phagocytic activity participates in fetal nutrition prior to the complete formation of the placenta [15]. The phagocytic activity also plays a role in acquiring space for embryo attachment and development in the endometrium [16].

Since it is thought that there are receptors against pathogens on TG cells, we attempted to identify the receptors and isolated a monoclonal antibody that inhibits bacterial uptake by TG cells [7]. The monoclonal antibody R2-25 reacted to heat shock cognate protein 70 (Hsc70). In the present study, we investigated the epitope of monoclonal antibody R2-25 on Hsc70 and showed that the binding of proteins containing tetratricopeptide repeat (TPR) domains to the C-terminal of Hsc70 through its EEVD motif inhibited bacterial uptake by TG cells.

## Methods

### Bacterial strains

*Brucella abortus *544 strain and *Listeria monocytogenes *EGD strain were used in this study. Bacterial strains were maintained as frozen glycerol stocks and cultured on Brucella broth or brain heart infusion (BHI) broth (Becton Dickinson, Franklin Lakes, NJ) or Brucella and BHI broth containing 1.5% agar.

### Cell culture

Trophoblast stem (TS) cells were cultured in TS medium in the presence of FGF4, heparin, and mouse embryonic fibroblast (MEFs)-conditioned medium as described previously [7]. The TS medium was prepared by adding 20% fetal bovine serum (FBS), 1 mM sodium pyruvate, 100 μM β-mercaptoethanol, and 2 mM L-glutamine to RPMI 1640. To induce differentiation to trophoblast giant (TG) cells, they were cultured in TS medium alone for 3 d at 37°C in a CO_2 _incubator. The TG cells were seeded (1-2 × 10^5 ^per well) in 48-well tissue culture plates for all assays.

### Preparation of recombinant proteins

Trigger factor (TF) fusion proteins of Hsc70, truncated Hsc70, TPR-Ba, and TPR-Lms tagged with six histidine residues at the N-terminus were constructed using the pCold TF system (Takara Bio, Shiga, Japan). pCold-Hsc70-I (1-646aa), pCold-Hsc70-II(1-484aa), pCold-Hsc70-III (162-646aa), pCold-Hsc70-IV (1-384aa), pCold-Hsc70-V (385-646aa), pCold-TPR-Ba, pCold-TPR-Lm1, and pCold-TPR-Lm2 were constructed by cloning PCR fragments into *Xho*I/*Sal*I-cleaved pCold TF vector. *hsc70-1*, *hsc70-II*, *hsc70-III*, *hsc70-IV*, *hsc70-V*, *tpr-Ba*, *tpr-Lm1 *or *tpr-Lm2 *were amplified by PCR using the primers *hsc70-I*, *hsc70-II*, and *hsc70-IV*: 5'-**CTCGAG**ATGTCTAAGGGACCTGCAGTT-3', *hsc70-III*: 5'-**CTCGAG**GGAACTATTGCTGGCCTCAAT-3', *hsc70-V*: 5'-**CTCGAG**TCTGAGAACGTTCAGGATTTG-3', *tpr-Ba*: 5'-**CTCGAG**ATGCTGCAATTGGCGATGCGC-3', *tpr-Lm1*: 5'-**CTCGAG**ATGCAAGAAGGTAATTTAGAA-3', or *tpr-Lm2*: 5'-**CTCGAG**ATGGAAAAAGACAAAAAAATA-3' (*Xho*I site underlined) and *hsc70-1*, *hsc70-III*, and *hsc70-V*: 5'-**GTCGAC**TTAATCCACCTCTTCAATGGT-3', *hsc70-II*: 5'-**GTCGAC**GCCATTGGCATCGATGTCAAA-3', *hsc70-IV*: 5'-**GTCGAC**CTTGTCTCCAGATAGAATGGC-3', *tpr-Ba*: 5'-**GTCGAC**TTAACCCCGCGTGCGGGCCAG-3', *tpr-Lm1*: 5'-**GTCGAC**TTACTCTGCTTCGTTTTCTAA-3', or *tpr-Lm2*: 5'-**GTCGAC**TTATCTGCTCAGGACTCGCTC-3' (*Sal*I site underlined). Each TF fusion protein was purified by Ni-NTA chromatography (Qiagen, Hilden, Germany).

### Immunoblotting

Protein samples were separated on 10% polyacrylamide gels and transferred to a PVDF membrane, which was incubated for 1 h at room temperature with anti-Hsc70 rat monoclonal antibody (SPA-815; Stressgen, Victoria, BC, Canada) at a dilution of 1:5000 in 5% skim milk. It was then washed three times in Tris-buffered saline (TBS) with 0.02% Tween 20, incubated for 30 min with a horseradish peroxidase (HRP)-conjugated secondary antibody at 0.01 μg/mL and washed again. Immunoreactions were visualized using the enhanced chemiluminescence detection system (GE Healthcare Life Science, Little Chalfont, UK).

### ELISA

Each synthetic peptide (10 μg/mL) was placed into 96-well immunoplates (Nalgene Nunc, Rochester, NY) and incubated at room temperature for 2 h. The sample was then removed, and the wells washed twice with phosphate buffered saline (PBS)-0.05% Tween 20. PBS containing 5% bovine serum albumin (BSA) was added to each well for blocking and incubated at 37°C for 30 min. A 100-μL aliquot of each R2-25 antibody (20 μg/mL) was added and the plate was incubated at 37°C for 2 h. After washing with PBS-0.05% Tween 20, HRP-conjugated anti-rat IgG was added and the plate was incubated at 37°C for 2 h. After washing as above, a substrate solution was added (1 mg/mL *p*-nitro phenyl phosphate in substrate buffer) (Sigma, St. Louis, MO). Absorbance was measured at 490 nm in a micro plate ELISA reader (Bio-Rad, Hercules, CA).

The ability of TPR proteins or bacterial cells to bind to Hsc70 was measured as follows. Aliquots of 100-μL TPR-Ba and TPR-Lms protein (10 μg/mL), or 10^8 ^heat-killed *B. abortus *and *L. monocytogenes *were placed into 96-well immunoplates and incubated at room temperature for 2 h. The sample was then removed, and the wells washed twice with PBS-0.05% Tween 20. PBS containing 1% BSA was added to each well for blocking prior to incubation at 37°C for 30 min. Aliquots of 100-μL Hsc70 (20 μg/mL) were added with or without TPR-Ba and TPR-Lms (10 μg/mL), and the plate was incubated at 37°C for 2 h. The amount of bound Hsc70 was determined by ELISA with anti-Hsc70 antibody.

### Efficiency of bacterial uptake by TG cells

Bacterial infection assays were performed according to the method of Watanabe *et al. *[7]. Bacterial strains were deposited onto TG cells at a multiplicity of infection of 100 which had been grown on 48-well microtiter plates containing TS medium but no antibiotics by centrifugation at 150 × *g *for 10 min at room temperature. To measure bacterial internalization efficiency after 30 min of incubation at 37°C, the cells were washed once with TS medium and then incubated with TS medium containing gentamicin (30 μg/mL) for 30 min. Next, cells were washed three times with PBS and lysed with cold distilled water. Colony-forming unit (CFU) values were determined by serial dilution on Brucella or BHI plates. The percentage protection was determined by dividing the number of bacteria surviving by the number in the infectious inoculum. The purified R2-25 antibody, recombinant TPR-Ba, and TPR-Lms proteins (at the indicated concentrations) were added to the TS medium 2 h before infection.

### Immunofluorescence microscopy

Bacteria were deposited onto TG cells grown on coverslips by centrifugation at 150 × *g *for 5 min at room temperature and were then incubated at 37°C for 30 min. Samples were washed twice with PBS and fixed with 4% paraformaldehyde in PBS for 30 min at room temperature. Subsequently, samples were washed three times in PBS and wells were successively incubated three times for 5 min in blocking buffer (5% BSA in PBS) at room temperature. Samples were stained with anti-*B. abortus *or anti-*L. monocytogenes *polyclonal rabbit serum diluted in blocking buffer (5 μg/mL) to identify extracellular bacteria. After incubating for 1 h at 37°C, samples were washed three times for 5 min with blocking buffer, stained with Cy5-labeled goat anti-rabbit IgG (0.01 μg/mL) (Chemicon, Temecula, CA) in blocking buffer, and incubated for 1 h at 37°C. Then, samples were permeabilized in 0.2% Triton X-100, and washed three times with PBS. The cells were incubated with anti-*B. abortus*, anti-*L. monocytogenes *and anti-Hsc70 (5 μg/mL) for 1 h at 37°C, and detected with FITC-labeled goat anti-rabbit IgG and TRITC-labeled goat anti-rat IgG (0.01 μg/mL) (Chemicon). Fluorescent images were obtained using a FluoView FV100 confocal laser scanning microscope (Olympus, Tokyo, Japan). Intracellular bacteria were detected by FITC, and the absence of staining with Cy5 [16]. A total of 100 TG cells were examined per coverslip to determine the number of intracellular bacteria.

### In vivo depletion of Hsc70

Groups of five pregnant mice were infected intraperitoneally with approximately 10^4 ^CFU of brucellae in 0.1 mL saline on day 4.5 of gestation [6]. Hsc70 was neutralized in the mice by administering an intravenous injection of anti-mouse Hsc70 monoclonal antibody (clone R2-25) or TPR-Ba using 100 μg (0.3 mL volume) intraperitoneally 24 h before infection. Control mice were given 100 μg of normal rat IgG (0.1 mL volume) according to the same injection schedule as used for the anti-Hsc70 monoclonal antibody and TPR-Ba inoculated groups. On day 18.5 of gestation, fetuses were removed from the mice and a judgment made as to whether they were pregnant or not. Fetuses were determined to be alive if there was a heartbeat, and dead if there was no heartbeat. EEVD or scrambled EEVD motif (sEEVD: CAPISEDGSGETV) mice were immunized intraperitoneally with Keyhole limpet hemocyanin (KLH) conjugated EEVD or sEEVD peptides (15 μg/each mouse) (Sigma) in PBS and the adjuvant (TiterMax Gold) (CytRx, Los Angeles, CA) at a ration of 1:1. Immunizations were performed 3 times at intervals of 2 weeks. The animal experiments were permitted by Animal Research Committee of Gyeongsang National University.

### Statistical analyses

Statistical analyses were performed using a Student's *t *test. Statistically significant differences compared with the control are indicated by asterisks (*, P < 0.01). Data are the averages of triplicate samples from three identical experiments, and the error bars represent standard deviations.

## Results

### Heat shock cognate protein 70 contributes to bacterial uptake by trophoblast giant cells

**Figure 1:**
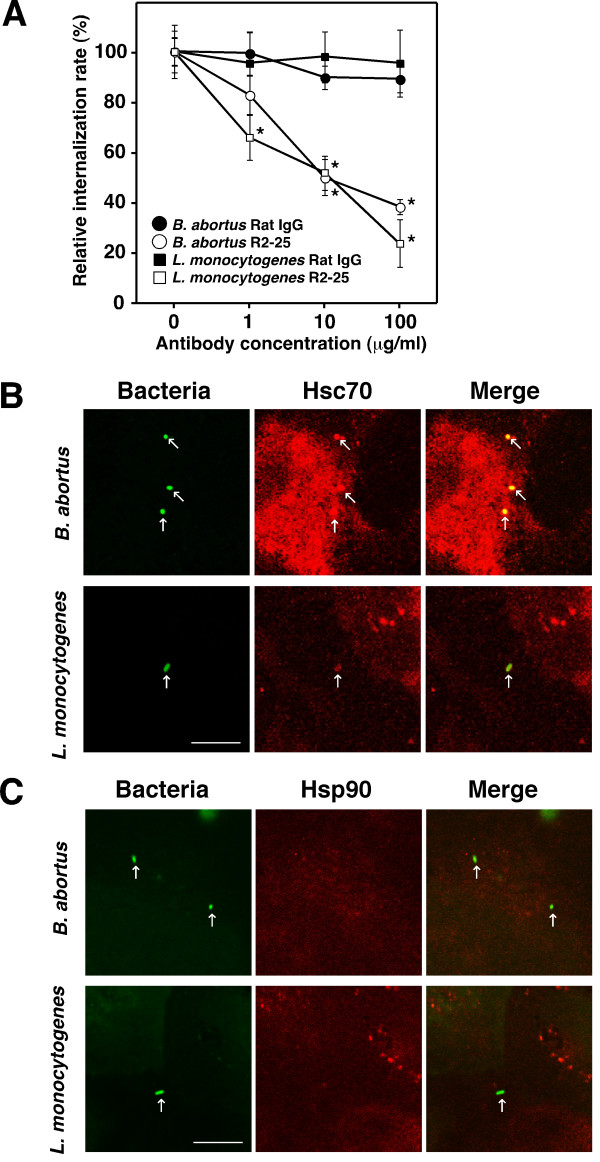
**Inhibition of bacterial internalization by the R2-25 antibody treatment**. (**A**) The purified R2-25 antibody was added to the cell culture medium for the bacterial internalization assay at the indicated concentrations 2 h before infection. Data are the averages of triplicate samples from three identical experiments, and the error bars represent standard deviations. Statistically significant differences between bacterial internalization into TG cells treated with the R2-25 antibody and those treated with rat IgG are indicated by asterisks (*, P < 0.01). Immunofluorescence staining of *B. abortus *or *L. monocytogenes *(green) and Hsc70 (**B**) or Hsp90 (red) (**C**) in TG cells. Colocalized bacteria with Hsc70 are shown in yellow in the merged images. Arrows point to colocalized bacteria. Scale bar indicates 10 μm.

In a previous study [7], we isolated a monoclonal antibody R2-25 that inhibits the uptake of *Brucella abortus *by trophoblast giant (TG) cells; this antibody reacts with heat shock cognate protein (Hsc70). Since Hsc70 on TG cells may be able to take up extracellular antigens such as bacteria, uptake of another abortion-inducing bacterium, *Listeria monocytogenes*, was examined. The purified R2-25 antibody significantly inhibited both *B. abortus *and *L. monocytogenes *uptake by TG cells concentration dependently, but there was no inhibition with rat IgG (negative control) (Fig. 1A). Hsc70 colocalized with *B. abortus *and *L. monocytogenes *at the site of bacterial uptake by TG cells (Fig. 1B), but the colocalization between Hsp90, other heat shock protein containing EEVD motif, and the bacteria was not detected (Fig. 1C).

### Antibody inhibiting bacterial uptake reacts with the EEVD motif of Hsc70

**Figure 2:**
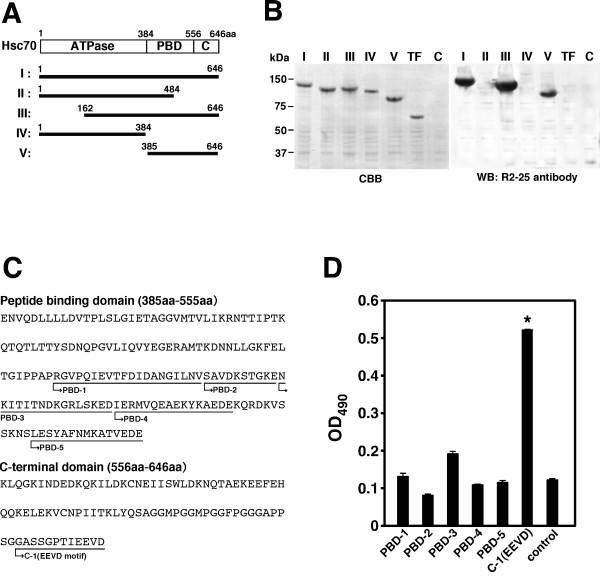
**Epitope mapping of monoclonal antibody R2-25**. (**A**) Schematic diagram of truncation constructs of Hsc70. A linear diagram of full-length Hsc70 is shown at the top; numbers indicate the corresponding amino acid position. The ATPase domain (ATPase), peptide binding domain (PBD), and C-terminal region (C) are shown. The truncation constructs generated are illustrated below and aligned corresponding to the full-length protein. The name of each construct is listed to the left of the illustration and all have an N-terminal trigger factor (TF). (**B**) Immunoblot analysis of Hsc70 truncation constructs. Purified proteins separated by SDS-PAGE and stained with Coomassie brilliant blue (CBB) (left panel), and the same samples were analyzed by immunoblotting with R2-25 antibody. TF, trigger factor; C, loading buffer. (**C**) Sequence of Hsc70 from 385 to 646 amino acids. Six synthesized peptides are shown underlined (PBD-1 to -5, and C-1). (**D**) Reaction activity of each synthetic peptide was measured by ELISA. Statistically significant differences between control (BSA) and C-1 (EEVD) peptides are indicated by asterisks (*, P < 0.01).

To identify the epitope of the R2-25 antibody on Hsc70, full length and four partial recombinant proteins were constructed (Fig. 2A). R2-25 reacted with the C-terminal region of Hsc70 (485-646 amino acids) (Fig. 2B). Next, the reaction of R2-25 with synthetic peptides containing 12-20 amino acids in this region was examined. Since R2-25 reacts with recombinant mouse and bovine Hsc70 [7], synthetic peptides were designed in homologous region between the mouse and bovine (Fig. 2C). It was found that R2-25 reacted with peptides representing the 12 C-terminal residues of Hsc70 containing the EEVD motif (Fig. 2D).

### Proteins containing the tetratricopeptide repeat domain bind to Hsc70 and inhibits bacterial uptake by TG cells

**Figure 3:**
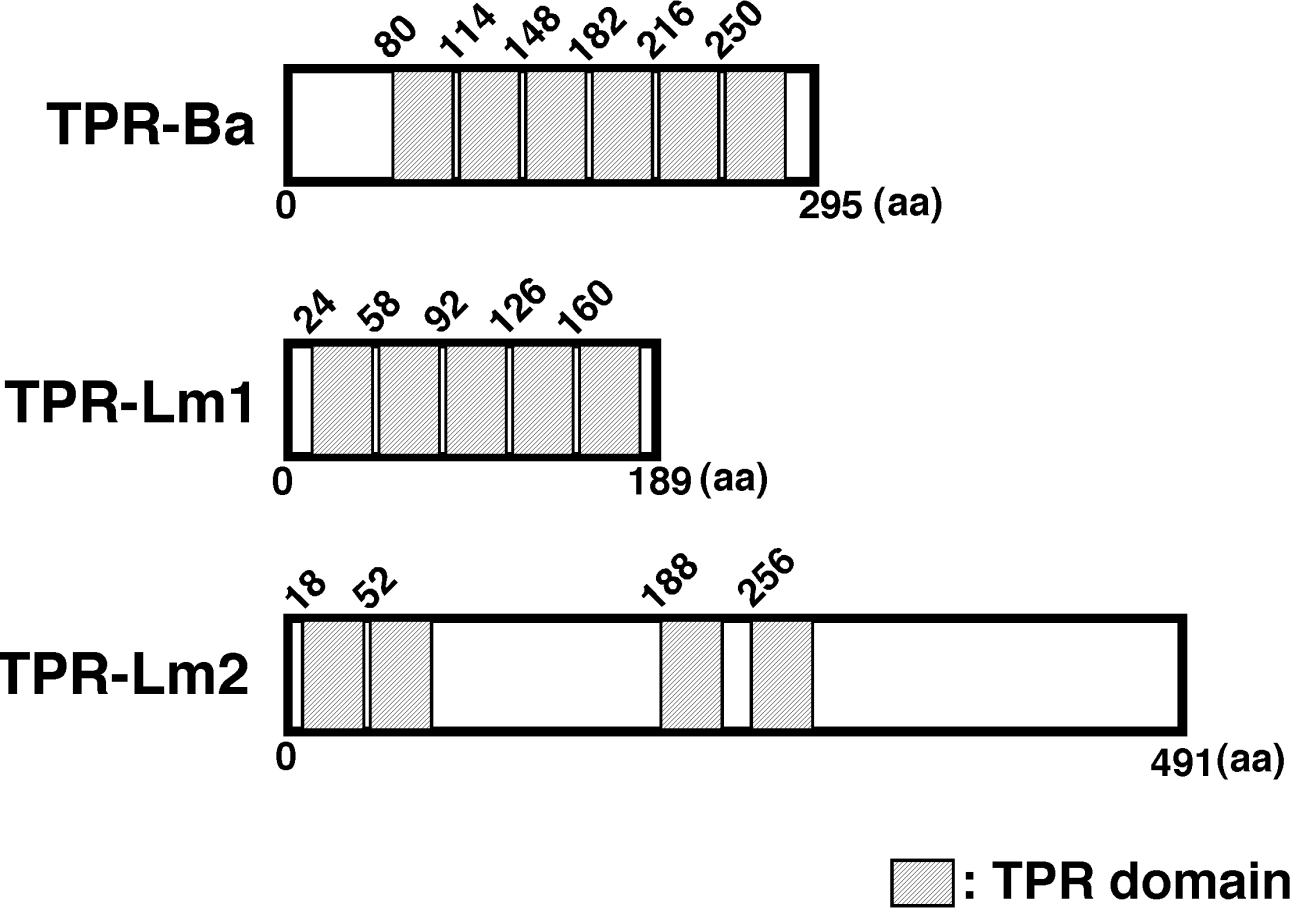
**The domain structures of TPR proteins**. TPR-Ba, TPR-Lm1, or TPR-Lm2 has six, five, or four TPR domains. TPR domain is indicated with striped bar. Domain search were performed using the SMART service http://smart.embl-heidelberg.de/.

Hsc70 has been reported to bind to proteins containing tetratricopeptide repeat (TPR) domains through its EEVD motif [17-19]. Therefore we hypothesized that bacterial proteins containing TPR domains could interact with Hsc70, and searched for such proteins in the genome database of *B. abortus *(GenBank AE017223, AE017224) and *L. monocytogenes *(GenBank AL591824). We found many TPR domain proteins in *B. abortus *and *L. monocytogenes*. In these TPR domain proteins, we selected a protein from *B. abortus *(BruAb2-0269, accession; YP_223064) and *L. monocytogenes *(Lmo-1510 and Lmo-2479, accession; NP_465035 and NP_466002) and designated them as TPR-Ba, TPR-Lm1, and TPR-Lm2, respectively. TPR-Ba and TPR-Lms (TPR-Lm1 and TPR-Lm2) contained six, five or four TPR domains (Fig. 3).

**Figure 4:**
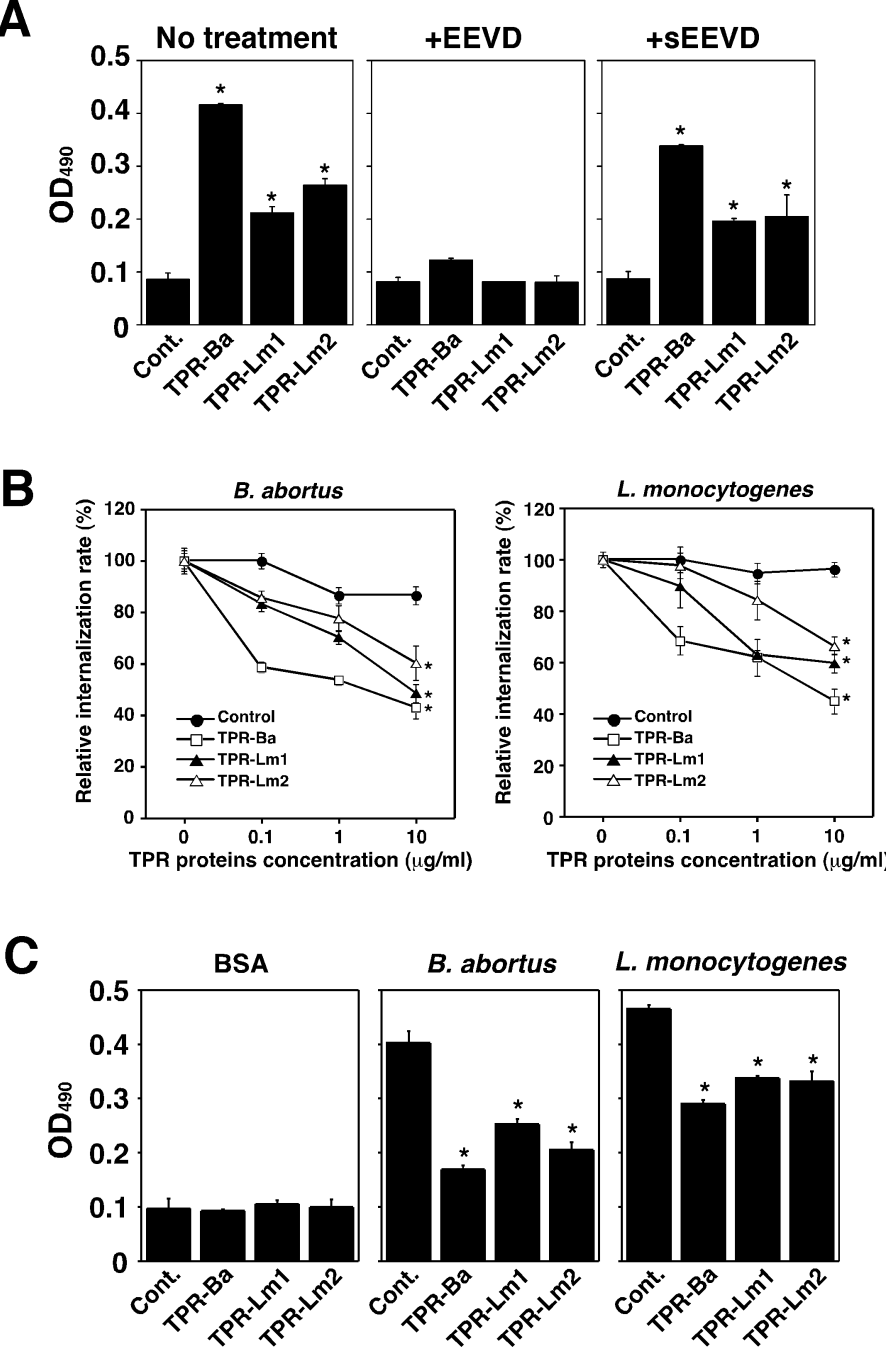
**Binding of TPR proteins to Hsc70**. (**A**) TPR proteins binding capacity for Hsc70 with or without EEVD or scrambled EEVD (sEEVD: CAPISEDGSGETV) peptides as measured by ELISA. Immunoplates were coated with TPR-Ba, TPR-Lm1, TPR-Lm2 proteins or BSA (Cont.) and then Hsc70 was added. Data are the averages of triplicate samples from three identical experiments, and the error bars represent standard deviations. Statistically significant differences between control and TPR proteins are indicated by asterisks (*, P < 0.01). (**B**) Interaction between TPR proteins and Hsc70 interferes with bacterial uptake by TG cells. Recombinant TPR proteins or BSA (Control) were added in the culture medium of TG cells at the indicated concentration and then bacteria were deposited onto TG cells. Data are the averages of triplicate samples from three identical experiments, and the error bars represent standard deviations. Statistically significant differences between control and TPR proteins are indicated by asterisks (*, P < 0.01). (**C**) *B. abortus *and *L. monocytogenes *binding capacity for Hsc70 with or without TPR proteins as measured by ELISA. Bacteria or BSA were coated on immunoplates and then TPR-Ba, TPR-Lm1, TPR-Lm2 proteins or BSA (Cont.) was added. After that, Hsc70 was added. Bacterial binding to Hsc70 were inhibited by addition of TPR proteins. Data are the averages of triplicate samples from three identical experiments, and the error bars represent standard deviations. Statistically significant differences between control and TPR proteins are indicated by asterisks (*, P < 0.01).

Interactions between Hsc70 and TPR domain proteins (TPR-Ba and TPR-Lms) through the EEVD motif were investigated. We tested TPR-Ba and TPR-Lms for their ability to bind to Hsc70 by means of ELISA with recombinant TPR-Ba, TPR-Lms, and Hsc70. The association was confirmed by ELISA using TPR-Ba or TPR-Lms-coated immunoplates. Hsc70 bound to the TPR-Ba or TPR-Lms-coated wells, and such binding was inhibited by the addition of peptides containing the EEVD motif, but not those containing the sEEVD (Fig. 4A).

Next, we studied the effect of adding TPR-Ba and TPR-Lms on bacterial uptake in a culture of TG cells. Treatment of TG cells with the TPR-Ba and TPR-Lms inhibited uptake of both *B. abortus *and *L. monocytogenes *by TG cells (Fig. 4B). TPR-Ba and TPR-Lms may negatively regulate the bacterial uptake function of Hsc70 on TG cells. To confirm this, the binding of Hsc70 to *B. abortus *and *L. monocytogenes *was assessed. Recombinant Hsc70 did bind to *B. abortus *and *L. monocytogenes*, and this was inhibited by TPR-Ba and TPR-Lms treatment (Fig. 4C).

### TPR protein inhibits infectious abortion

**Figure 5:**
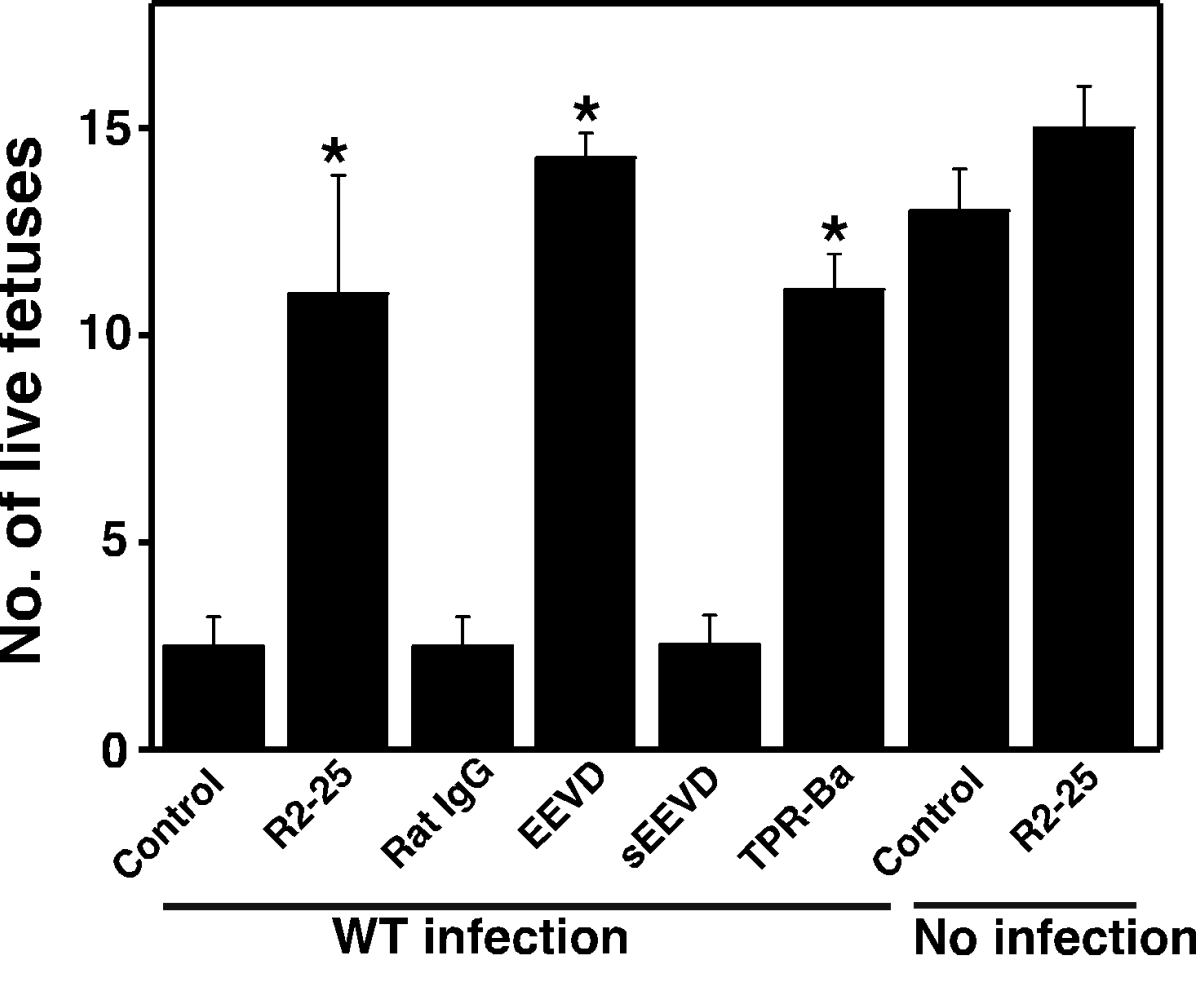
**Control of infectious abortion by protein interaction via the EEVD motif of Hsc70**. Infectious abortion was prevented by blocking the EEVD motif of Hsc70. Pregnant mice were inoculated with R2-25, rat IgG (control), TPR-Ba and immunized with EEVD or sEEVD (CAPISEDGSGETV) peptides. Control received no treatment. Statistically significant differences between the untreated control and antibody treated mice are indicated by asterisks (*, P < 0.01).

We finally investigated the effect on pregnancy by blocking the interaction between Hsc70 and bacteria in pregnant mice. Pregnant mice were inoculated with R2-25 or TPR-Ba, evaluated as the most effective TPR protein according to the results above, 24 h before infection with the wild-type *B. abortus *strain on day 4.5 of gestation. While abortions were observed in the non-inoculated and Rat IgG inoculated mice, there was a significant increase in the number of live fetuses in the R2-25 or TPR-Ba inoculated mice. Abortion was also inhibited by immunization using a peptide containing the EEVD motif before infection (Fig. 5).

## Discussion

Phagocytic activity of TG cells has been observed in the presence of lipopolysaccharide (LPS) or IFN-γ [7,20]. TG cells are polyploid cells that play a crucial role in implantation, remodeling of the embryonic cavity, and preventing maternal blood flow to the implantation site [21]. Furthermore, the phagocytic activity of TG cells participates in fetal nutrition prior to complete formation of the placenta and plays a role in embryo attachment and development in the endometrium. TG cells are also able to phagocytize microorganisms, supporting the hypothesis that a possible trophoblast defense mechanism contributes to the removal of infectious agents from the fetal-maternal interface [13]. The uptake activity of bacteria by TG cells was inhibited by adding TPR-Ba or TPR-Lms. These results suggest that the phagocytosis of TG cells using surface-presented Hsc70 could form an embryonic and fetal innate immune defense through elimination of the microorganisms present at the maternal-fetal interface. Hsc70 has been reported to be present on the surface of several types of cells, and can bind fatty acids and bacterial LPS [22,23]. We recently reported that ezrin, a member of the family of ezrin-radixin-moesin (ERM) proteins, associated with Hsc70, and that Hsc70 may be able to present on membranes by interacting with ezrin in TG cells [24]. It has also been suggested that extracellular Hsp70 and Hsc70 possess immunological properties and they are commonly perceived as being inflammatory mediators [25]. Thus, surface-presented Hsc70 on TG cells would recognize bacterial molecules such as LPS or components of the bacterial cell wall and phagocytize microorganisms lysing the cells. Since the amino acid sequences of human and mouse Hsc70 are identical, the function of their Hsc70 is assumed to be the same. Rodent TG cells are analogous to extravillous cytotrophoblast cells of the human placenta, both are polyploid and invasive, and have similar patterns of trophoblast cell subtype-specific gene expression [26].

The TPR domain is a protein-protein interaction motif that was originally identified through sequence comparisons among yeast proteins [27]. A number of TPR containing proteins participate in interactions with major members of the heat shock protein family, *i.e*., Hsp70, Hsc70, and Hsp90, and are necessary for the appropriate regulation of protein folding and transport [28]. Further, one of the TPR containing proteins, the carboxyl terminus of Hsc70-interacting protein (CHIP), negatively regulates chaperone activities of Hsc70 [29]. Hsc70 binds to bacterial molecules in the uptake of extracellular pathogens, and the binding of TPR-Ba and TPR-Lms to the C-terminal of Hsc70 would also negatively regulate this bacterial uptake function of Hsc70 on TG cells. The anti-Hsc70 monoclonal antibody that inhibited bacterial uptake by TG cells mimics the interaction between TPR containing proteins and the C-terminal of Hsc70. We have previously made TPR-Ba deletion mutant in *B. abortus *and investigated the virulence of the mutant. Although we expected that deletion mutant failed to invade into TG cells, and exhibited weak virulence in mouse, the results were contrary (data not shown). The mutant showed hypervirulence because of loss of TPR proteins as a negative regulator. However, it also remains possible that deletion of TPR-Ba may change the property of some molecules and affect virulence indirectly.

In previous studies, we showed that *B. abortus *replicated preferentially in TG cells in the placenta [6,7]. Trophoblastic cells are the early targets of *L. monocytogenes *[10]. Since abortion-inducing bacteria such as *B. abortus *and *L. monocytogenes *infect TG cells and replicate in them, the cell functions are suppressed to some extent, which leads to abortion since implantation and placental development are inhibited. Therefore, it is thought that bacterial infection of TG cells is a key event in inducing abortion. In our previous study [7], abortion by *B. abortus *infection was inhibited in pregnant mice that were inoculated with the R2-25 antibody 24 h before bacterial infection. This suggests that infectious abortion could be prevented by blocking bacterial uptake by TG cells.

## Conclusion

The results in this study show that the bacterial uptake function of Hsc70 is inhibited by binding between proteins containing TPR domains, TPR-Ba and TPR-Lms, and the C-terminal of Hsc70 through the EEVD motif. We could not find any typical transmembrane domains, secretion signals, or lipid anchor sequence in TPR-Ba and TPR-Lms. However unknown transmembrane sequence or unconventional secretion systems independent on signal sequence would contribute to distribution of the TPR proteins on bacterial surface or extracellular fractions. Alternatively, it is possible that TPR proteins might be released from damaged or destroyed bacteria and they interact with Hsc70 on cell surface. Although the function of TPR-Ba and TPR-Lms in bacterial physiology is still unknown, these molecules could be useful in the development of methods for preventing infectious abortion.

## Competing interests

The authors declare that they have no competing interests.

## Authors' contributions

MW conceived the study. MW and KW designed the experiments, interpreted the results, and worked on the manuscript. KW and MT carried out most of the experimental works. KS participated in cell culture and experiments of infection. All authors read and approved the final manuscript.
